# Cardiac telocytes were decreased during myocardial infarction and their therapeutic effects for ischaemic heart in rat

**DOI:** 10.1111/j.1582-4934.2012.01655.x

**Published:** 2012-12-04

**Authors:** Baoyin Zhao, Shang Chen, Juanjuan Liu, Ziqiang Yuan, Xufeng Qi, Junwen Qin, Xin Zheng, Xiaotao Shen, Yanhong Yu, Thomas J Qnin, John Yeuk-Hon Chan, Dongqing Cai

**Affiliations:** a Key Laboratory for Regenerative Medicine, Ministry of Education, Ji Nan UniversityGuangzhou, China; b Joint Laboratory for Regenerative Medicine, Chinese University of Hong Kong-Ji Nan UniversityGuangzhou, China; c International Base of Collaboration for Science and Technology (JNU), The Ministry of Science and Technology & Guangdong ProvinceGuangzhou, China; d Department of Developmental and Regenerative Biology, Ji Nan UniversityGuangzhou, China; e Department of Molecular Genetics, Albert Einstein College of MedicineNew York, USA

**Keywords:** cardiac telocytes, myocardial infarction, regeneration

## Abstract

Recently, cardiac telocytes were found in the myocardium. However, the functional role of cardiac telocytes and possible changes in the cardiac telocyte population during myocardial infarction in the myocardium are not known. In this study, the role of the recently identified cardiac telocytes in myocardial infarction (MI) was investigated. Cardiac telocytes were distributed longitudinally and within the cross network of the myocardium, which was impaired during MI. Cardiac telocytes in the infarction zone were undetectable from approximately 4 days to 4 weeks after an experimental coronary occlusion was used to induce MI. Although cardiac telocytes in the non-ischaemic area of the ischaemic heart experienced cell death, the cell density increased approximately 2 weeks after experimental coronary occlusion. The cell density was then maintained at a level similar to that observed 1–4 days after left anterior descending coronary artery (LAD)-ligation, but was still lower than normal after 2 weeks. We also found that simultaneous transplantation of cardiac telocytes in the infarcted and border zones of the heart decreased the infarction size and improved myocardial function. These data indicate that cardiac telocytes, their secreted factors and microvesicles, and the microenvironment may be structurally and functionally important for maintenance of the physiological integrity of the myocardium. Rebuilding the cardiac telocyte network in the infarcted zone following MI may be beneficial for functional regeneration of the infarcted myocardium.

## Introduction

Despite the initial optimism for the use of extra cardiac stem cells in the regeneration of the infarcted myocardium, reconstruction of the damaged myocardium remains challenging [1–4]. During development and under pathological conditions, adult interstitial cells and microvessels control the proliferation, growth and differentiation of cardiomyocytes [4–7]. Thus, we should consider a new perspective in which all cells of the myocardium are important for tissue homeostasis, development, disease and regeneration [8, 9]. Therefore, understanding how cardiac cells work together to maintain structural and functional integrity in disease and regeneration is critical for elucidating the cellular and molecular mechanisms of regeneration following myocardial infarction. Such knowledge could also provide a starting point for the development of new therapies for regeneration following myocardial infarction (MI).

Recently, a novel type of interstitial cell termed telocytes was found in the interstitium of the heart [10–17], intestine [18], uterus and fallopian tube [19], trachea and lung [20, 21], skeletal muscle [22], mammary gland [23] and placenta [24]. Within the cardiac stem cell niche, cardiac telocytes play an essential role as niche-supporting cells that nurse the cardiac stem cells and angiogenic cells in the myocardium. Furthermore, they may play an important role during regeneration following MI [25]. However, the exact role of the cardiac telocytes in the myocardium is still not clear. Understanding the possible changes that cardiac telocytes undergo during MI may elucidate their functional role during homeostasis, disease and regeneration of the myocardium. Previously, we reported the distribution of cardiac telocytes in the subepicardium and endocardium in the atrium-atria, medium and base parts of the heart. The density of cardiac telocytes in the base and the atrium-atria parts of the heart was significantly higher than that in the medium part of the heart. In addition, the density of cardiac telocytes in the subepicardium was significantly higher than that in the endocardium [26]. These findings suggest that cardiac telocytes may play an important role in the maintenance of the structural and functional integrity of the myocardium. In this study, we propose to investigate possible changes in the distribution of cardiac telocytes within the heart after myocardial infarction. We also propose a method for transplantation of cardiac telocytes into the infarcted heart to facilitate regeneration following MI.

## Materials and methods

### Animals

Three-month-old female Sprague-Dawley (SD; 250–300 g) rats were utilized in this study. The rats were housed for at least 2 weeks before being used for experiments with food and water provided *ad libitum*. Animal care, surgery and handling procedures were performed according to the guidelines of The Ministry of Science and Technology of the People's Republic of China [(2006)398] and approved by the Ji Nan University Animal Care Committee.

### Myocardial infarction studies

Series of 3-month-old female SD rats (*n* = 3 for each group) were utilized to establish myocardial infarction as previously described [27]. Briefly, the rats were anesthetized with ketamine (100 mg/kg i.p.) prior to undergoing a left intercostal thoracotomy. After the left anterior descending coronary artery (LAD) was identified, the LAD was ligated directly below the left atrial appendage with 8-0 nylon sutures. Abnormality in the pallor and regional wall motion of the left ventricle confirmed the occlusion. The chest wall was then closed, the lungs were inflated, the rat was extubated and the tracheotomy was closed. After recovery, the rats were returned to the animal facility for 1–28 days. The ligated hearts were harvested at different time intervals after LAD-ligation (1, 2, 3, 4, 5, 6, 7, 14 and 28 days) and embedded in OCT compound (Sakura Finetek, Torrance, USA). Consecutive frozen sections (10 μm) were collected for each whole heart and prepared for immuofluorescent staining. For histology staining, the ligated hearts were fixed in 4% paraformaldehyde and embedded in paraffin. The sections were stained using Masson's trichrome staining.

### Immuofluorescent staining

Representative sections of the atrium-atrial segments, the medium segment and the base segment of the individual hearts (Fig. 2IIa) were used for immuofluorescent staining. For double immuofluorescent staining, after washing three times with PBS (pH = 7.4), rabbit anti-rat c-kit antibody (1:300; cat. no. NBP1-19865; Novus, Littleton, USA) was added to the sections, and the sections were incubated at 4°C overnight. Then, FITC-donkey anti-rabbit IgG was added, and the sections were incubated for 30 min. Subsequently, goat anti-rat-CD34 antibody (1:300; cat. no. ZDP0111041; R&D, Minneapolis, USA) was added to the sections, and the sections were incubated again at 4°C overnight. Texas Red-donkey anti-goat IgG was then added, and the sections were incubated for 30 min. The sections were then counter-stained with DAPI and mounted with mounting medium. In the above procedure, three PBS (pH = 7.4) washes were conducted after each step. For semi-quantitative analysis of the cardiac telocytes in the representative sections of the atrium-atria, the medium and the base parts of the heart, rabbit anti-rat c-kit antibody (1:300; cat. no. NBP1-19865; Novus) was added to the sections and the sections were incubated at 4°C overnight. Cy3-goat anti-rabbit IgG was then added and the sections were incubated for 30 min. Finally, the slides were counter-stained with DAPI and mounted with mounting medium. Three PBS (pH = 7.4) washes were conducted after each step in the above procedure.

## 3D-reconstruction of cardiac telocytes in the myocardium

The sections that were immuofluorescently stained for c-kit were digitized using a Peltier-cooled digital monochrome CCD camera (Photometrics-RT100361, Roper Scientific, Trenton, USA) attached to an API image system (Applied Precision Inc, WA, USA) with Z-scan. Imaris software (Version 7.4; Bitplane, Zurich, Switzerland) was used to create 3D-reconstructions and to analyse the sections. All the single-slice image frames were merged and then saved as a file with a.tif format. All the images were deconvoluted with 3D-blind deconvolution using the 3D-constructor function with the image voxel size set to auto, a sub sampling size of 1 × 1 × 1, a transparency of 0.747 and with the ‘palette’ acting. The parameters of opaque, brightness, contrast, gamma and 3D-filter were modified accordingly. After the 3D-reconstruction, the iso-surface of the reconstructed subject was created, and the diameter and length were measured.

### Semi-quantification of cardiac telocytes

Currently, no specific biomarkers exist for the identification of cardiac telocytes. However, cell morphology and positive expression of c-kit have been used as a standard for the identification of cardiac telocytes [10]. In this study, we used the positive expression of c-kit with a DAPI-positive nucleus and a very small cell body (piriform/spindle/triangular) with extremely long and thin prolongation (length ≥60 μm) to identify cardiac telocytes. Images (40×) of the infarcted zone (five images in the central area), the border zone (10 images on each side) and the zone opposite the infarction zone (five images opposite from the position of the central infarction zone in the left ventricle wall; shown as a schematic in Fig. 2IIb) were obtained using fluorescence microscopy (Wetzlar GmbH- DM4000B; Leica, Solms, Germany) as representative areas of each part (the base, the medium and the atrium-atria parts of the heart; as shown in the schematic in Fig. 2IIa). The anti-c-kit and DAPI images from the same field were merged using Leica cw4000 FISH software. The cardiac telocytes were counted using a double-blinded method. The cell density in mm^2^ ± the SD was used for the semi-quantitative analysis.

### Isolation of cardiac telocytes

Young (3-month-old) female SD rats were used for the isolation of cardiac telocytes. The mini-magnetically activated cell sorting system (Miltenyi Biotec, Cologne, Germany) was used to sort c-kit^+^ cardiac telocytes. The hearts of the rats were removed and minced in DMEM (Gibco, NY, USA) containing 1% penicillin and streptomycin (Gibco), and then the hearts were digested with 2.5 ml of DMEM+ 0.05% collagenase P (Roche, Indianapolis, USA) and 0.1% trypsin (Amresco, Solon, USA) at 37°C with shaking (180 r.p.m.) for 15 min. After the suspension was removed, digestion medium was added and the mixture was incubated at 37°C with shaking (180 r.p.m.) for 45 min. The digested tissue was dissociated by pipetting gently every 15 min. The supernatant was then filtered through 100- and 41-μm nylon mesh (Millipore, MA, USA), and the collected suspension was centrifuged at 50 × *g* for 2 min. The supernatant was then removed and re-centrifuged at 300 × *g* for 10 min. The supernatant was discarded, and the pellet was re-suspended in 5 ml of PEB medium [PBS supplemented with 0.5% bovine serum albumin and 2 mM EDTA (pH = 7.2)]. The mixture was then centrifuged at 38 × *g* for 2 min. to remove the debris, and the collected supernatant was further centrifuged at 200 × *g* for 10 min. The cell pellet was then mixed with 1 ml of PEB and 5 μl of rabbit anti-rat c-kit antibody (1:200; cat. no. NBP1-19865; Novus), and the sample was incubated at 4°C for 40 min. An additional 2 ml of PEB was then added, and the mixture was centrifuged at 458 *g* for 4 min. to collect the cells. The pellet was re-suspended in 160 μl of PEB, and 20 μl of a solution containing magnetic beads (goat anti-rabbit lgG-microbeads, cat. no. 5111007039; Miltenyi Biotec) was added, followed by incubation at 4°C for 25 min. The mixture was next added to an MC column (Miltenyi Biotec) in a magnetic field, and the unlabelled cells were allowed to pass through. The MC column was then removed from the magnetic field, and the labelled cells were flushed out with PEB. The isolated cell pellet was collected after centrifugation at 1600 r.p.m. for 4 min.

### Purity of isolated cardiac telocytes

The purity of the isolated cardiac telocytes was determined by double immuofluorescent staining for c-kit and CD34. Isolated cardiac telocytes (10^4^) were cultured on a coverslip and fixed with 4% paraformaldehyde. After washing three times with PBS (pH = 7.4), rabbit anti-rat c-kit antibody (1:300; cat. no. NBP1-19865; Novus) was added and the samples were incubated at 4°C overnight. FITC-donkey anti-rabbit IgG was then added, and the samples were incubated for 30 min. Goat anti-rat-CD34 antibody (1:300; cat. no. ZDP0111041; R&D) was then added, and the samples were incubated again at 4°C for 12 hrs again. The Texas Red-donkey anti-goat IgG was added, and incubated for 30 min. After that, the cells were counter-stained with DAPI and mounted with mounting medium. During the above procedure, three PBS (pH = 7.4) washes were conducted for each step. For the semi-quantitative analysis of purity, 20 fields (20×) were randomly captured using a fluorescent microscope (Olympus-IX51 with DP72-CCD, Olympus Corporation, Tokyo, Japan). The percentage of cells that were c-kit^+^, CD34^+^ or c-kit^+^ and CD34^+^ was determined. Three animals were applied in this part study.

### Transplantation of cardiac telocytes

To study the potential therapeutic effects of cardiac telocytes in MI, series of young female SD rats (3 months old; *n* = 5 for the cardiac telocyte-injected group; *n* = 9 for the c-kit negative cell injection group; *n* = 8 for the PBS control group) were used to establish myocardial infarction as previously described [20]. The intra-myocardial injections were performed within 30 min. of the LAD-ligation. One million cardiac telocytes in PBS, 10^6^ c-kit negative cells in PBS or PBS only were injected through a 30-gauge needle using a 100-μl Hamilton syringe. Five injections (10 μl/injection; three injections in the border area of the ischaemic zone and two injections in the centre of the ischaemic area) were administered. The chest wall was then closed, the lungs were inflated, the rat was extubated, and the tracheotomy was closed. After recovery, the rats were returned to the animal facility for 14 days. The extent of myocardial infarction was measured at the level of the midpapillary heart muscles and scored by Masson's trichrome staining. The images were analysed using Image J 1.22 software (NIH Image, WA, USA). The infarction size, with linear approximations to account for missing areas in histology, was expressed as a percentage of the total left ventricle myocardial area as previously described [27]. Two investigators independently performed the quantification in a blinded manner.

### Echocardiography

Transthoracic echocardiograms were recorded in rats 14 days after LAD-ligation and injection of cardiac telocytes. The rats (*n* = 5 for the cardiac telocyte injection group; *n* = 9 for the c-kit negative cell injection group; *n* = 8 for the PBS control group; *n* = 5 for the sham group; *n* = 5 for the normal control group) were anesthetized with ketamine (100 mg/kg bw, i.p.). The echocardiographic parameters were then measured using an Acuson Sequoia 256c equipped with a 13-MHz linear transducer Vevo 770 echocardiogram (VisualSonics, Toronto, Canada). The anterior chest was shaved, and the rats were placed in the left lateral decubitus position. A rectal temperature probe was placed, and the body temperature was carefully maintained between 37.0 and 37.5°C using a heating pad throughout the study. The parasternal long-axis, parasternal short-axis and two apical four-chamber views were used to obtain 2D-M-mode. The systolic and diastolic anatomical parameters were obtained from M-mode tracings at the midpapillary level. The ejection fraction (EF) was calculated using the area-length method [28, 29].

### Statistical analysis

All data are presented as the means ± SD. A one-way (anova) and the LSD test were applied using SPSS version-11. *P* < 0.05 was considered statistically significant.

## Results

### Identification of cardiac telocytes

**Fig. 1:**
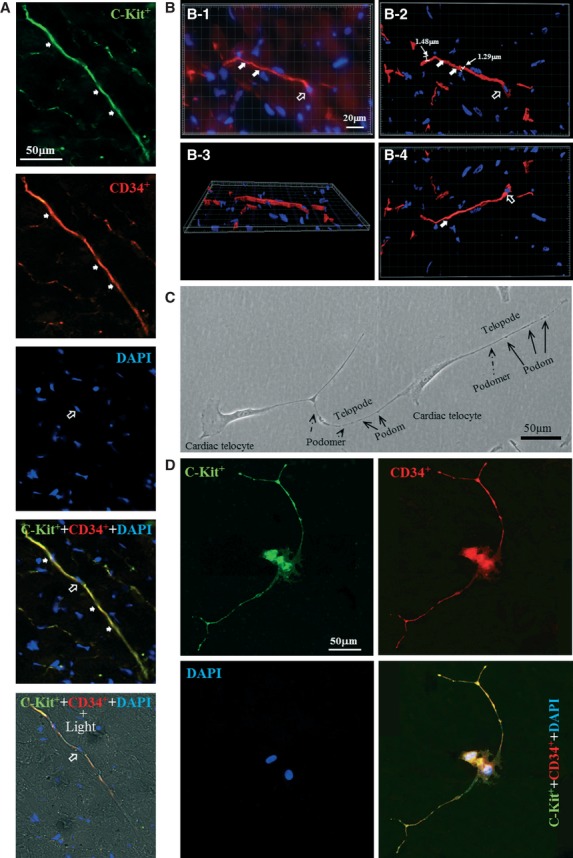
Identification of cardiac telocytes. (**A**) Double immuofluorescent staining for anti-c-kit (green) and CD34 (red) demonstrated c-kit^+^ and CD34^+^ cells with very small cell bodies (approximately 1:1 ratio of the cytoplasm to the nucleus; open arrow) and extremely thin prolongation in the interstitial space of cardiac myocytes. Some dilation was found in the thin prolongation of the c-kit^+^ and CD34^+^ cells (arrow). (**B**) 3D-recontruction of a representative cell further confirmed that the cell consisted of a small cell body with a nucleus (approximately 1:1 ratio of the cytoplasm to the nucleus; open arrow) and an extremely thin prolongation (diameter: approximately 1–2 μm; arrow). (B-1) representative *Z*-axis image. (B-2) 3-D reconstruction of B-1 with all *Z*-axis images. (B-3) 90° rotation of the *X*-axis of the images shown in B-2. (B-4) 180° rotation of the *X*-axis of the image shown in B-2. (**C**) Primary culture of isolated cardiac telocytes revealed that under phase-contrast microscopy, cardiac telocytes displayed piriform/spindle/triangular cell bodies and long, slender telopodia that contain alternations of the thick segments, *i.e*. podoms (arrow), and thin segments, *i.e*. podomers (dotted line arrow). (**D**) Cardiac telocytes with unique morphology are c-kit^+^ and CD34^+^. *n* = 3 for each group.

**Fig. 2:**
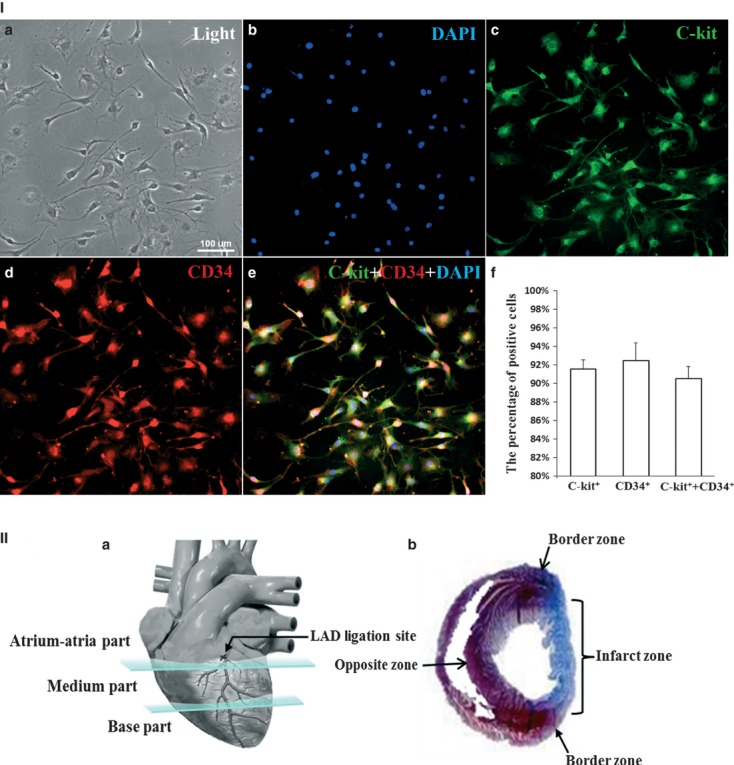
Purity of the isolated cardiac telocytes and schematic sections of the heart. (**I**) Double immunofluorescent staining for anti-c-kit and CD34 combined with cell counting demonstrated that approximately 91.5 ± 0.01% of the cells were c-kit positive, approximately 92.5 ± 0.02% of the cells were CD34 positive and approximately 90.5 ± 0.01% of the cells were c-kit and CD34 positive. a: light; b: DAPI; c: anti-c-kit (green); d: anti-CD34 (red); e: merged images from b, c and d. f: quantification of cells that were positive for c-Kit, CD34 and c-Kit and CD34 (*n* = 3). (**II**) a: schematic representation of the atrium-atria, medium and base parts of the heart; b: schematic representation of the infarcted zone, border zone and the zone opposite the infarcted zone.

Double immuofluorescent staining for anti-c-kit and anti-CD34 in sections from the whole heart demonstrated many c-kit^+^ and CD34^+^ cells with very small cell bodies (approximately 1:1 ratio for the cytoplasm and nucleus) and extremely thin prolongation in the interstitial space of the cardiac myocytes. In addition, the cell prolongations had a moniliform aspect (alternations and dilated segments; Fig. 1A). 3D-recontruction of one cell confirmed that it had a very small cell body with a nucleus (approximately 1:1 ratio of the cytoplasm to the nucleus) and one extremely long prolongation (diameter of approximately 1–2 μm) with some dilation (Fig. 1B_1-4_). Furthermore, under phase-contrast microscopy, the primary culture of isolated c-kit^+^ cardiac telocytes revealed cardiac telocytes with piriform/spindle/triangular cell bodies containing long and slender telopods, which present the alternation of thick segments –podoms and thin segments – podomers (Fig. 1C). These cardiac telocytes with unique morphology were positive for expression of c-kit and CD34 (Fig. 1D). The purity of the isolated cardiac telocytes was determined using double immunofluorescent staining for anti-c-kit and CD34. Approximately 91.5 ± 0.01% of the cells were c-kit positive, approximately 92.5 ± 0.02% of the cells were CD34 positive and approximately 90.5 ± 0.01% of the cells were c-kit and CD34 positive (Fig. 2I).

### Distribution of the cardiac telocytes in the myocardium

**Fig. 3:**
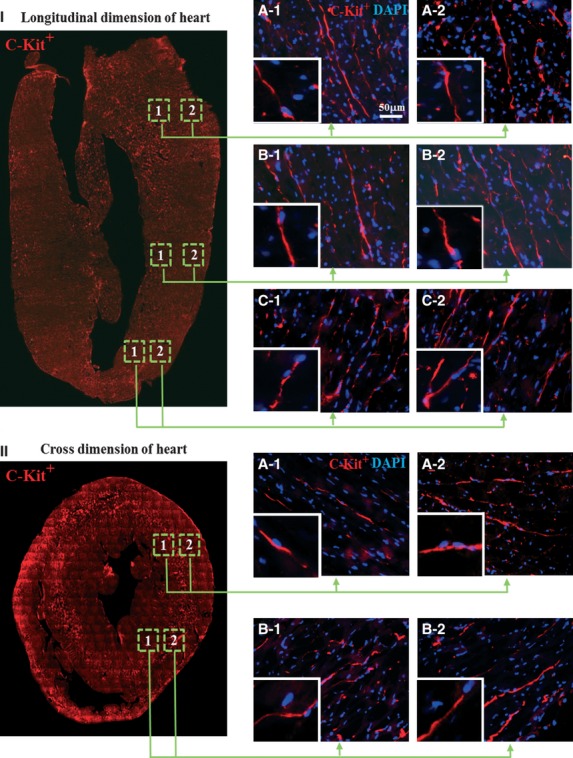
Distribution of cardiac telocytes in the myocardium. (**I**) Immuofluorescent staining for c-kit (red) revealed that many of the cardiac telocytes were distributed in the longitudinal dimension of the whole heart (A1-2, B1-2 and C1-2). (**II**) All of the cardiac telocytes were distributed within the cross direction (A1-2 and B1-2). Figures inset in I-A1-2, I-B1-2, I-C1-2, II-A1-2 and II-B1-2 contain images of cardiac telocytes at a higher magnification (*n* = 3).

Nearly all the c-kit^+^ cells with a very small cell body (piriform/spindle/triangular) and extremely thin prolongation were also CD34^+^. Therefore, this unique morphology, which is characterized by a small piriform/spindle/triangular cell body containing a DAPI^+^ nucleus and extremely thin prolongation (length ≥60 μm), and c-kit expression were used as the standard to identify and quantify cardiac telocytes in the myocardium. Anti-c-kit immuofluorescent staining of the longitudinal dimension of the whole heart demonstrated that many cardiac telocytes were distributed within the longitudinal direction (Fig. 3I). Similarly, in the sample of the entire cross dimension that was stained with anti-c-kit, many cardiac telocytes were distributed within the cross direction (Fig. 3II).

### Decrease in cardiac telocytes during MI

#### Semi-quantification of cardiac telocytes in the atrium-atria part of the heart

**Fig. 4:**
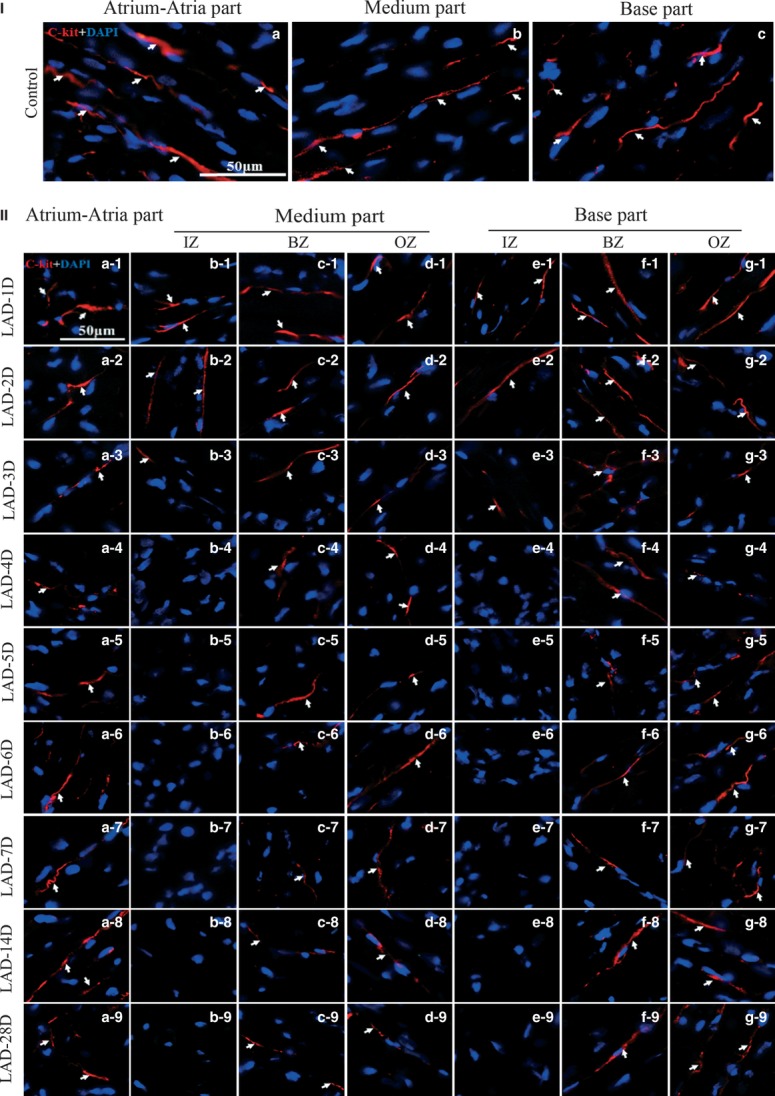
Comparison of cardiac telocytes in different segments of heart during MI. (**I**) Showing the cardiac telocyte density of table-1 in same region in the non-LAD ligated control of atrium-atria part (a), medium part (b) and base part (c). (**II**) Showing the change in cardiac telocyte density of table-1 for atrium-atria part of MI (a1-9); Showing the change in cardiac telocyte density of table-1 for infarcted zone of medium part of MI (b1-9); Showing the change in cardiac telocyte density of table-1 for border zone of medium part of MI (c1-9); Showing the change in cardiac telocyte density of table-1 for zone opposite the infarcted zone of medium part of MI (d1-9); Showing the change in cardiac telocyte density of table-1 for infarcted zone of base part of MI (e1-9); Showing the change in cardiac telocyte density of table-1 for border zone of base part of MI (f1-9); Showing the change in cardiac telocyte density of table-1 for zone opposite the infarcted zone of base part of MI (g1-9). IZ: infarcted zone. BZ: border zone. OZ: zone opposite the infarcted zone. *n* = 3.

In the atrium-atria part of the heart, the density of cardiac telocytes progressively decreased 1–4 days after LAD-ligation as compared to the same region in the non-LAD ligated control group. The density of cardiac telocytes was lowest 3–4 days after LAD-ligation and increased progressively 5–28 days after LAD-ligation (*P* < 0.05). At 14–28 days after LAD-ligation, the density of cardiac telocytes was maintained at a level similar to that measured 1 day after LAD-ligation (Table 1 and Fig. 4Ia and IIa1-9).

**Table 1:** Comparison of cardiac telocytes in different segments of heart during MI

		Medium part of the heart			Base part of the heart		
			
	Atrium-atria	Infarcted zone	The border zone	The opposite zone	Infarcted zone	The border zone	The opposite zone
Con	41.03 ± 1.5	28.57 ± 1.3	30.04 ± 2.2	30.04 ± 1.6	44.69 ± 0.7	43.96 ± 1.4	44.69 ± 1.4
LAD-1 day	33.70 ± 1.7	20.51 ± 1.7*	27.84 ± 1.4	29.30 ± 2.7	23.44 ± 2.1*	41.76 ± 1.9	42.49 ± 1.0
LAD-2 days	22.71 ± 2.6*	13.92 ± 3.1*	24.91 ± 1.9	25.64 ± 1.2	14.65 ± 2.9*	35.16 ± 2.1*	32.23 ± 1.5*
LAD-3 days	18.32 ± 4.2*	5.86 ± 4.1 *	22.71 ± 1.2	19.05 ± 2.4*	5.86 ± 3.0*	25.64 ± 0.8*	22.71 ± 2.7*
LAD-4 days	18.32 ± 2.8*	ND	21.52 ± 0.8*	19.78 ± 3.1*	2.19 ± 4.3*	23.44 ± 1.1*	22.71 ± 1.3*
LAD-5 days	22.44 ± 1.4*	ND	17.58 ± 1.8*	21.98 ± 1.2*	ND	21.98 ± 1.9*	27.11 ± 0.6*
LAD-6 days	25.64 ± 2.6*	ND	16.12 ± 3.3*	24.18 ± 1.2	ND	21.25 ± 2.4*	30.04 ± 2.3*
LAD-7 days	30.04 ± 3.6*	ND	13.92 ± 2.8*	24.18 ± 2.3	ND	21.25 ± 1.7*	32.97 ± 0.4*
LAD-14 days	32.97 ± 2.7*	ND	21.25 ± 2.9*	26.37 ± 2.3	ND	24.18 ± 2.9*	32.97 ± 1.5*
LAD-28 days	32.97 ± 1.4*	ND	21.98 ± 0.9*	26.37 ± 0.8	ND	24.18 ± 2.1*	32.97 ± 0.5*

* *P* < 0.05; *versus* same region in the non-LAD ligated control. Con: same region in the non-LAD ligated control. ND: nondetectable.

#### Semi-quantification of the cardiac telocytes in the medium part of the heart

In the medium part of the heart, the density of cardiac telocytes in the infarcted zone, the border zone of the infarcted site and opposite the infarcted zone was investigated (Fig. 2IIb). In the infarcted zone, the density of cardiac telocytes decreased progressively compared with the same region in the non-LAD ligated control group 1–3 days after LAD-ligation and these cells were undetectable 4 days after LAD-ligation (*P* < 0.05; Table 1 and Fig. 4Ib and IIb1-9).

In the border zone, the density of cardiac telocytes decreased significantly 4–7 days after LAD-ligation when compared with the same region in the non-LAD ligated control group (*P* < 0.05). Furthermore, the density of cardiac telocytes decreased progressively from day 1 to day 7 after LAD-ligation and reached its lowest level 7 days after LAD-ligation. However, the telocyte density increased slightly by 14 days after LAD-ligation and was then maintained at a level similar to that observed 4 days after LAD-ligation (*P* < 0.05; Table 1 and Fig. 4Ib and IIc1-9).

In the zone located opposite the infarcted zone, the density of cardiac telocytes decreased significantly 3–4 days after LAD-ligation when compared with the same region in the non-LAD ligated control group (*P* < 0.05). The decrease in the density of cardiac telocytes was progressive 2–4 days after LAD-ligation, and the telocyte density reached its lowest point 3–4 days after LAD-ligation (*P* < 0.05). However, the density of cardiac telocytes increased 5 days after LAD-ligation and was then maintained at a level similar to that measured 2 days after LAD-ligation (Table 1 and Fig. 4Ib and IId1-9).

#### Semi-quantification of cardiac telocytes in the base part of the heart

In the base part of the heart, the density of cardiac telocytes in the infarcted zone, the border zone of the infarcted site and the zone opposite the infarcted zone was measured (Fig. 2IIb). In the infarcted zone, the density of cardiac telocytes decreased progressively 1–4 days after LAD-ligation when compared with the same region in the non-LAD ligated control group, and the cells became undetectable 5 days after LAD-ligation (*P* < 0.05; Table 1 and Fig. 4Ic and IIe1-9).

In the border zone, the density of cardiac telocytes decreased significantly 2–28 days after LAD-ligation when compared with the same region in the non-LAD-ligated control group (*P* < 0.05). The density of cardiac telocytes decreased progressively from 2 to 7 days after LAD-ligation and reached its lowest level approximately 6–7 days after LAD-ligation. However, the density increased slightly by day 14 after LAD-ligation and was then maintained at a level similar to that observed 4 days after LAD-ligation (*P* < 0.05; Table 1 and Fig. 4Ic and IIf1-9).

In the zone opposite the infarcted zone, the density of cardiac telocytes decreased significantly 2–28 days after LAD-ligation when compared with the same region in the non-LAD-ligated control group (*P* < 0.05). The density of cardiac telocytes decreased progressively for 2 days after LAD-ligation and reached its lowest level approximately 3–4 days after LAD-ligation. However, the density increased by day 5 after LAD-ligation and was then maintained at a level similar to that observed 2 days after LAD-ligation (*P* < 0.05; Table 1 and Fig. 4Ic and IIg1-9).

### Transplantation of cardiac telocytes into the ischaemic myocardium decreased infarction size and improved myocardial function

**Fig. 5:**
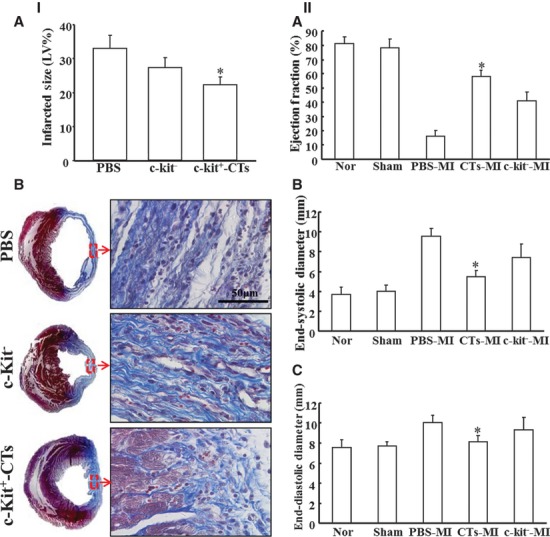
Transplantation of cardiac telocytes decreased infarction size and improved myocardial function following MI. Cardiac telocytes (10^6^) were simultaneously injected into the infarcted zone and the border zone. The infarction size was analysed 14 days after LAD ligation. (**I**-A) The infarction size (%LV) of the cardiac-telocyte-injected group was significantly lower than that of the c-kit-negative cell-injected and PBS-injected control groups (*P* < 0.05). However, the infarction size (%LV) of the c-kit-negative cell-injected control group was similar to the infarction size in the PBS control group (*P* > 0.05). *P < 0.05; cardiac-telocyte-injected group *versus* c-kit-negative cell-injected control group or cardiac-telocyte-injected group *versus* PBS-injected control group. (I-B) Masson's trichrome staining revealed that in the cardiac-telocyte-injected hearts, some cardiac myocytes survived in the infarcted zone; however, the zones were primarily composed of fibrotic tissue in the c-kit-negative cell-injected and PBS-injected control groups. (**II**) Functional assay of MI *via* echocardiography with a 2D-M-model demonstrated that the ejection fraction (EF) of the CT-treated group was significantly higher than that of the c-kit-negative cell-injected and the PBS-injected control groups (*P* < 0.05). However, it was still significantly lower than that in the sham and normal control groups (*P* < 0.05). *P < 0.05; the cardiac telocyte-MI group *versus* all other groups (A). The end-systolic diameter and the end-diastolic diameter of the cardiac-telocyte-injected group were lower than those of the c-kit-negative cell-injected and PBS-injected control groups (*P* < 0.05 *versus* the PBS control group), but higher than that of the sham and the normal control groups (*P* < 0.05; B and C). *P < 0.05; the cardiac telocyte-MI group *versus* PBS control group, sham group and normal control group. *n* = 5 for the cardiac-telocyte-injected group; *n* = 9 for the c-kit-negative cell-injected group; *n* = 8 for the PBS control group; *n* = 5 for the sham group; *n* = 5 for the normal control group. CTs: cardiac telocytes. Nor: normal control.

The therapeutic effect of injecting cardiac telocytes into the heart during MI was also investigated. Cardiac telocytes (10^6^) were simultaneously injected into the infarcted zone and the border zone after MI. The infarction size was analysed 14 days after LAD-ligation. The infarction size (%LV) in the cardiac-telocyte-treated group was lower than that in the c-kit-negative cell-treated and PBS-treated control groups (*P* < 0.05). However, the infarction size in the group that received the c-kit-negative cells was similar to that of the PBS-treated control group (*P* > 0.05; Fig. 5I-A). In addition, Masson's trichrome staining revealed that in the cardiac-telocyte-injected hearts, some cardiac myocytes survived in the infarcted zone, whereas the infarcted zone was mainly composed of fibrotic tissue in the c-kit-negative cell-injected and PBS-injected control groups (Fig. 5I-B). Echocardiography in the MI hearts revealed that the EF of the cardiac-telocyte-treated group was significantly higher than that of the c-kit-negative cell-treated and PBS-treated control groups (*P* < 0.05). However, the EF was still significantly lower than that of the sham and the normal control groups (*P* < 0.05; Fig. 5II-A). The final systolic and diastolic diameters of the cardiac-telocyte-treated group were lower than those of the c-kit-negative cell-treated and PBS-treated control groups (*P* < 0.05 *versus* PBS-treated group). However, they were higher than those of the sham and the normal control groups (*P* < 0.05; Fig. 5II-B and II-C).

## Discussion

Cardiac telocytes have been found in the myocardium, epicardium and endocardium and in cardiac stem cell niches [10–17, 25, 26, 30]. Previously, we reported that the density of cardiac telocytes in the base and atrium-atria parts of the heart was significantly higher than that in the medium part. In addition, the density of cardiac telocytes in the subepicardium was significantly higher than that in the endocardium [26]. This finding suggests that cardiac telocytes may play an important role in the maintenance of the structural and functional integrity of the myocardium. In this study, we further found that cardiac telocytes are distributed longitudinally and within the cross network in normal myocardium. We also demonstrate that the number of cardiac telocytes decreases and that the cardiac telocyte network is destroyed during myocardial infarction.

Four or five days after LAD-ligation, cardiac telocytes in the infarcted zone were undetectable in the medium and base parts of the heart. This phenomenon was not unexpected because cardiac telocytes in this area were undergoing cell death because of ischaemia. Cardiac telocytes were also undetectable in the infarcted zones of these two parts of the heart until 4 weeks after LAD-ligation. These results suggest that the ischaemic microenvironment of the infarcted zone does not support live cardiac telocytes approximately 4–5 days after myocardial infarction. Furthermore, cardiac telocytes in the border zone of the myocardial infarction or from other exogenous sources failed to migrate into the infarction zone, which could be a critical pathological change that occurs following myocardial infarction, but one that has not been previously described. This pathology may be related to the poor healing and regeneration of the heart that is observed following myocardial infarction. Therefore, it may be possible to disturb the structural and functional network consisting of cardiac telocytes, myocytes, microvascular endothelial cells and endogenous cardiac stem cells. Indeed, recent studies have reported that cardiac telocytes establish direct nanocontact with myocytes as well as *de novo* contact with the endothelial cells of the border zone of the MI and with endogenous cardiac stem cells. Furthermore, it has been reported that cardiac telocytes release vesicles and/or exosomes [16, 17, 25] and that they express VEGF and NOS2, and contain measurable angiogenic microRNAs. Therefore, cardiac telocytes may contribute to neo-angiogenesis within the myocardium *via* paracrine effects [16]. Our transplantation of cardiac telocytes during MI also demonstrated that simultaneous transplantation of cardiac telocytes into both the central and border zones of the myocardial infarction after ischaemia decreased the infarction size and improved myocardial function. This result was confirmed by an increased EF and a decreased end-systolic and end-diastolic diameter 2 weeks after transplantation. To the best of our knowledge, this is the first report to show that after MI, transplantation of cardiac telocytes decreases infarction size and improves myocardial function in ischaemic rat hearts. Therefore, our findings suggest that the cardiac telocyte network and its secreted factors and microvesicles may be important structural and functional factors that maintain the function of the myocardium. Thus, rebuilding the cardiac telocyte network through transplantation or by inducing the migration of cardiac telocytes into the infarction zone following MI may result in functional regeneration of the infarcted myocardium.

In addition, cardiac telocytes were undetectable in the infarcted zone of the medium and base parts of the heart approximately 4–5 days after LAD-ligation. However, in the atrium-atria and the border and the zone opposite the medium and base parts, the cardiac telocyte density progressively increased from 5 to 7 days after LAD-ligation and then (until 28 days after LAD-ligation) maintained a density similar to that observed 1–4 days after LAD-ligation. This finding suggests that ischaemia in the myocardium initiated the damaging effects in the whole myocardium and induced the death of cardiac telocytes. However, it appears that the ischaemic microenvironment still allowed the survival of cardiac telocytes in the non-ischaemic areas, but not in the infarcted zone. Furthermore, the proliferation phase of infarction healing occurs 48 hrs to 5 days after occlusion in rodents, and during this phase, the fibroblasts and endothelial cells are activated and proliferate [31–33]. Therefore, the lack of detection of cardiac telocytes within the infarcted zone 4–5 days after occlusion of LAD-ligation indicates that cardiac telocytes may be more susceptible to death than fibroblasts and endothelial cells in the ischaemic microenvironment of the myocardium. This phenomenon may be a unique parameter that defines cardiac telocytes functionally, which indicates that it is important to develop an effective method to maintain the survival of cardiac telocytes during coronary occlusion to protect the ischaemic myocardium and regenerate the infarcted myocardium.

## References

[1] Bernstein HS, Srivastava D (2012). Stem cell therapy for cardiac disease. Pediatr Res.

[2] Leon MP, Moussa M, Jeremy NR (2012). Towards regenerative therapy for cardiac disease. The Lancet.

[3] Segers VF, Lee RT (2008). Stem-cell therapy for cardiac disease. Nature.

[4] Ieda M, Tsuchihashi T, Ivey KN (2009). Cardiac fibroblasts regulate myocardial proliferation through beta1 integrin signaling. Dev Cell.

[5] Thum T, Gross C, Fiedler J (2008). MicroRNA-21 contributes to myocardial disease by stimulating MAP kinase signaling in fibroblasts. Nature.

[6] Narmoneva DA, Vukmirovic R, Davis ME (2004). Endothelial cells promote cardiac myocyte survival and spatial reorganization. Circulation.

[7] Zaglia T, Dedja A, Candiotto C (2009). Cardiac interstitial cells express GATA4 and control dedifferentiation and cell cycle re-entry of adult cardiomyocytes. J Mol Cell Cardiol.

[8] Ausoni S, Sartore S (2009). The cardiovascular unit as a dynamic player in disease and regeneration. Trends Mol Med.

[9] Anderson RH, Smerup M, Sanchez-Quintana D (2009). The three-dimensional arrangement of the myocytes in the ventricular walls. Clin Anat.

[10] Popescu LM, Faussone-Pellegrini MS (2010). TELOCYTES — a case of serendipity: the winding way from interstitial cells of cajal (ICC), *via* interstitial cajal-like cells (ICLC) to telocytes. J Cell Mol Med.

[11] Popescu LM, Manole CG, Gherghiceanu M (2010). Telocytes in human epicardium. J Cell Mol Med.

[12] Bani D, Formigli L, Gherghiceanu M (2010). Telocytes as supporting cells for myocardial tissue organization in developing and adult heart. J Cell Mol Med.

[13] Kostin S (2010). Myocardial telocytes: a specific new cellular entity. J Cell Mol Med.

[14] Gherghiceanu M, Manole CG, Popescu LM (2010). Telocytes in endocardium: electron microscope evidence. J Cell Mol Med.

[15] Gherghiceanu M, Popescu LM (2011). Heterocellular communication in the heart: electron tomography of telocyte-myocyte junctions. J Cell Mol Med.

[16] Manole CG, Cismaşiu V, Gherghiceanu M (2011). Experimental acute myocardial infarction: telocytes involvement in neo-angiogenesis. J Cell Mol Med.

[17] Gherghiceanu M, Popescu LM (2012). Cardiac telocytes - their junctions and functional implications. Cell Tissue Res.

[18] Carmona IC, Bartolome MJ, Escribano CJ (2011). Identification of telocytes in the lamina propria of rat duodenum: transmission electron microscopy. J Cell Mol Med.

[19] Popescu LM, Ciontea SM, Cretoiu D (2007). Interstitial Cajal-like cells in human uterus and fallopian tube. Ann NY Acad Sci.

[20] Zheng Y, Li H, Manole CG (2011). Telocytes in trachea and lungs. J Cell Mol Med.

[21] Popescu LM, Gherghiceanu M, Suciu LC (2011). Telocytes and putative stem cells in the lungs: electron microscopy, electron tomography and laser scanning microscopy. Cell Tissue Res.

[22] Popescu LM, Manole E, Serboiu CS (2011). Identification of telocytes in skeletal muscle interstitium: implication for muscle regeneration. J Cell Mol Med.

[23] Gherghiceanu M, Popescu LM (2005). Interstitial Cajal-like cells (ICLC) in human resting mammary gland stroma. Transmission electron microscope (TEM) identification. J Cell Mol Med.

[24] Suciu L, Popescu LM, Gherghiceanu M (2010). Telocytes in human term placenta: morphology and phenotype. Cells Tissues Organs.

[25] Gherghiceanu M, Popescu LM (2010). Cardiomyocyte precursors and telocytes in epicardial stem cell niche: electron microscope images. J Cell Mol Med.

[26] Liu JJ, Shen XT, Zheng X (2011). Distribution of telocytes in the rat heart. J Clin Rehabil Tiss Eng Res.

[27] Cai D, Holm JM, Duignan IJ (2006). BDNF-mediated enhancement of inflammation and injury in the aging heart. Physiol Genomics.

[28] Dawn B, Stein AB, Urbanek K (2005). Cardiac stem cells delivered intravascularly traverse the vessel barrier, regenerate infarcted myocardium, and improve cardiac function. Proc Natl Acad Sci USA.

[29] Linke A, Müller P, Nurzynska D (2005). Stem cells in the dog heart are self-renewing, clonogenic and multipotent and regenerate infarcted myocardium, improving cardiac function. Proc Natl Acad Sci USA.

[30] Faussone-Pellegrini MS, Bani D (2010). Relationships between telocytes and cardiomyocytes during pre- and post-natal life. J Cell Mol Med.

[31] Dobaczewski M, Bujak M, Zymek P (2006). Extracellular matrix remodeling in canine and mouse myocardial infarcts. Cell Tissue Res.

[32] Dobaczewski M, Gonzalez-Quesada C, Nikolaos GF (2010). The extracellular matrix as a modulator of the inflammatory and reparative response following myocardial infarction. J Mol Cell Cardiol.

[33] Dewald O, Ren G, Duerr GD (2004). Of mice and dogs: species-specific differences in the inflammatory response following myocardial infarction. Am J Pathol.

